# Preventing Disused Bone Loss through Inhibition of Advanced Glycation End Products

**DOI:** 10.3390/ijms24054953

**Published:** 2023-03-03

**Authors:** Cong-Jin Liu, Xiao Yang, Shou-Hui Wang, Xin-Tong Wu, Yan Mao, Jing-Wen Shi, Yu-Bo Fan, Lian-Wen Sun

**Affiliations:** Key Laboratory for Biomechanics and Mechanobiology, Ministry of Education, Beijing Advanced Innovation Center for Biomedical Engineering, School of Biological Science and Medical Engineering, Beihang University, Beijing 100083, China

**Keywords:** simulated microgravity, bone loss, advanced glycation end products, irbesartan

## Abstract

Bone loss occurs in astronauts during long-term space flight, but the mechanisms are still unclear. We previously showed that advanced glycation end products (AGEs) were involved in microgravity-induced osteoporosis. Here, we investigated the improvement effects of blocking AGEs formation on microgravity-induced bone loss by using the AGEs formation inhibitor, irbesartan. To achieve this objective, we used a tail-suspended (TS) rat model to simulate microgravity and treated the TS rats with 50 mg/kg/day irbesartan, as well as the fluorochrome biomarkers injected into rats to label dynamic bone formation. To assess the accumulation of AGEs, pentosidine (PEN), non-enzymatic cross-links (NE−xLR), and fluorescent AGEs (fAGEs) were identified in the bone; 8-hydroxydeoxyguanosine (8-OHdG) was analyzed for the reactive oxygen species (ROS) level in the bone. Meanwhile, bone mechanical properties, bone microstructure, and dynamic bone histomorphometry were tested for bone quality assessment, and Osterix and TRAP were immunofluorescences stained for the activities of osteoblastic and osteoclastic cells. Results showed AGEs increased significantly and 8-OHdG expression in bone showed an upward trend in TS rat hindlimbs. The bone quality (bone microstructure and mechanical properties) and bone formation process (dynamic bone formation and osteoblastic cells activities) were inhibited after tail-suspension, and showed a correlation with AGEs, suggesting the elevated AGEs contributed to the disused bone loss. After being treated with irbesartan, the increased AGEs and 8-OHdG expression were significantly inhibited, suggesting irbesartan may reduce ROS to inhibit dicarbonyl compounds, thus suppressing AGEs production after tail-suspension. The inhibition of AGEs can partially alter the bone remodeling process and improve bone quality. Both AGEs accumulation and bone alterations almost occurred in trabecular bone but not in cortical bone, suggesting AGEs effects on bone remodeling under microgravity are dependent on the biological milieu.

## 1. Introduction

Significant losses of weight-bearing bones occur in astronauts during long-term space flight [1]. Specifically, about 1–1.6% of bone mineral in the spine, femur neck, trochanter, and pelvis can be lost per month; elevated calcium excretion also occurs in astronauts during spaceflight [2,3]. However, the prolonged microgravity-induced bone loss cannot be reversed for a long time after the flight and recovery times are much longer than even the duration of the mission [1,4,5]. Previous studies reported the countermeasures to prevent microgravity-induced bone loss such as physical exercise and drug supply only showed limited efficiency [6,7]. That is because the mechanism of microgravity-induced osteoporosis is still unclear.

Recently, our study found non-enzymatic compounds called advanced glycation end products (AGEs) accumulated significantly in the bone matrix under simulated microgravity [8]. These non-enzymatic compounds are formed through the irreversible rearrangements of Amadori products which are formed via a series of reactions of reducing sugars (e.g., blood glucose or ribose) and amino groups in long-lived proteins (e.g., collagen), or through the condensation of amino groups and dicarbonyl compounds, which are derived from the autooxidation of sugars and Amadori products [9]. Indeed, the accumulation of AGEs has been found to be associated with an increased risk of fractures in individuals with age-related and diabetic osteoporosis [10,11,12,13,14,15,16]. AGEs were preserved in old trabeculae and altered the final architecture of trabecular bone and decreased bone toughness in age-related osteoporosis [15,17]. Moreso, they impaired bone microarchitecture, as well as bone mechanical properties, including toughness, energy absorption, yield strength, and failure load, further leading to bone fracture in diabetic osteoporosis [18,19,20,21]. Moreover, on cellular level, AGEs can interact with the receptor for AGEs (RAGE) to suppress osteoblast growth and differentiation and impair mineralization [22,23,24,25]. However, their modulation of osteoclast activity is controversial [26,27,28,29], and some researchers proposed it may be because AGEs had biphasic modulation during the different differentiation stages of osteoclasts [30].

Given the adverse impact of AGEs on bone, more and more studies focused on the use of AGEs inhibitors for improving bone quality and the bone remodeling process under pathological conditions. Previous studies have found that cleaving formed AGEs cross-linking structures can decrease bone fragility in vitro [31,32]. Moreover, blocking the interaction between AGEs with RAGE can rescue the negative effects of AGEs on osteogenic potential of adipose-derived stem cells (ASCs) and affect osteoblast differentiation/activity in an age-dependent manner [33,34]. However, directly blocking AGEs formation at the root can attenuate the deterioration of bone in more ways because it not only prevented the biomechanical degradation of bone and inhibited AGEs-induced damage on cell proliferation and osteogenic differentiation of osteoblasts in vitro, but also was applied on in vivo animal experiments to improve both trabecular microstructure and bone toughness of diabetic mice [35,36].

Therefore, we wish to know that under simulated microgravity, whether blocking AGEs formation can also prevent its accumulation to stop the occurrence of disused bone loss. Of all the inhibitors that prevent the formation of AGEs, Angiotensin II receptor blockers (ARBs) are unique in that they do not trap dicarbonyl precursors for AGEs formation. Instead, they directly inhibit the formation of reactive oxygen species such as carbon-centered and hydroxyl radicals, which in turn suppresses the auto-oxidation of sugars into dicarbonyl precursors. As a result, ARBs are considered more effective than other inhibitors [37]. Irbesartan, as one of the ARBs, was commonly used to lower blood pressure [38]. However, more recently, it has been found in vivo to exhibit therapeutic effects on bone microarchitecture and biomechanical properties in diabetic mice (e.g., ultimate tensile strength, max load, fracture load, and energy absorption) [36].

In order to investigate the issue we mentioned above, we treated tail-suspended rats with irbesartan for 3 weeks at a concentration of 50 mg/kg/day in this study. Three methods were used to evaluate AGEs accumulation in bone matrix. Meanwhile, bone microstructure parameters, bone micromechanical properties, dynamic bone formation, and the activities of osteoblastic and osteoclastic cells were detected. Their relationships with the content of AGEs were analyzed as well. The objectives of this study were (1) to provide detailed information on the relationship between disused bone loss and AGEs, and (2) to determine the effects of irbesartan on matrix AGEs accumulation in bone under simulated microgravity and whether blockage of AGEs formation can alleviate the microgravity-induced bone loss.

## 2. Results

### 2.1. Irbesartan Inhibited AGEs Accumulation in Bone Matrix after Tail Suspension

**Figure 1:**
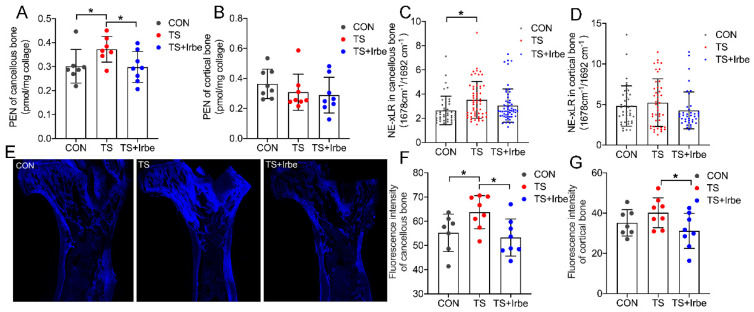
**Irbesartan inhibited AGEs accumulation in bone matrix after tail suspension.** (**A**) PEN content of cancellous bone. (**B**) PEN content of cortical bone. (**C**) NE−xLR in cancellous bone. (**D**) NE−xLR in cortical bone. I Spontaneous fluorescence images of bone slices. (**E**) spontaneous fluorescence images. (**F**) Fluorescence intensity of cancellous bone. (**G**) Fluorescence intensity of cortical bone. Values are all expressed as mean ± SD. * *p* < 0.05.

Here, we first analyzed PEN content, a standard biomarker for AGEs in bone tissue [38], in different types of bone in rat hindlimb quantitatively. The HPLC results showed that PEN content of cancellous bone obviously increased (Figure 1A, *p* < 0.05) after tail suspension, but significantly decreased with irbesartan treatment (TS + Irbe) compared to the TS group (*p* < 0.05). However, that of cortical bone showed no significant difference in TS and TS + Irbe group compared to the CON group (Figure 1B).

Further at the micro level in the bone matrix, we analyzed NE−xLR by FTIR and fAGEs content by fluorescence microscopy, respectively. The results showed that in cancellous bone NE−xLR (Figure 1C) and fAGEs (Figure 1F) increased significantly (*p* < 0.05) in the TS group, but fAGEs decreased significantly with irbesartan treatment in the TS + Irbe group (*p* < 0.05). In cortical bone (Figure 1D,G), NE−xLR had no significant difference in the TS and TS + Irbe groups compared to the CON group, whereas fAGEs decreased significantly in the TS + Irbe group compared to the TS group (*p* < 0.05). These results indicated that AGEs accumulation in the bone matrix, especially in trabecular bone after tail suspension, was inhibited with irbesartan treatment.

### 2.2. Irbesartan Can Inhibit Reactive Oxygen Species in Bone Matrix after Tail Suspension

**Figure 2:**
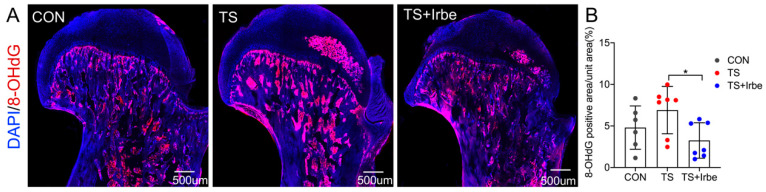
**Irbesartan can inhibit reactive oxygen species in bone matrix after tail suspension.** (**A**) Representative images of 8-OHdG immunofluorescence staining (red), Nuclei was stained with DAPI (blue). (**B**) 8-OHdG positive area per unit area (%). * *p* < 0.05.

Because Irbesartan was found to suppress reactive oxygen species (ROS) to inhibit the generation of dicarbonyl compounds, thus suppressing AGEs production [37], here we detected 8-hydroxydeoxyguanosine (8-OHdG), which is a kind of ROS marker, by immunofluorescence staining. The results showed (Figure 2) that 8-OHdG in the bone matrix showed an upward trend in the TS group compared to the CON group, whereas it decreased significantly with the drug treatment in the TS + Irbe group, compared to the TS group (*p* < 0.05). These results suggested that irbesartan may suppress reactive oxygen species to inhibit dicarbonyl compounds generation, thus inhibiting the production of AGEs in the bone matrix after tail suspension.

### 2.3. Inhibition of AGEs Partially Improved Bone Microstructure after Tail Suspension

**Figure 3:**
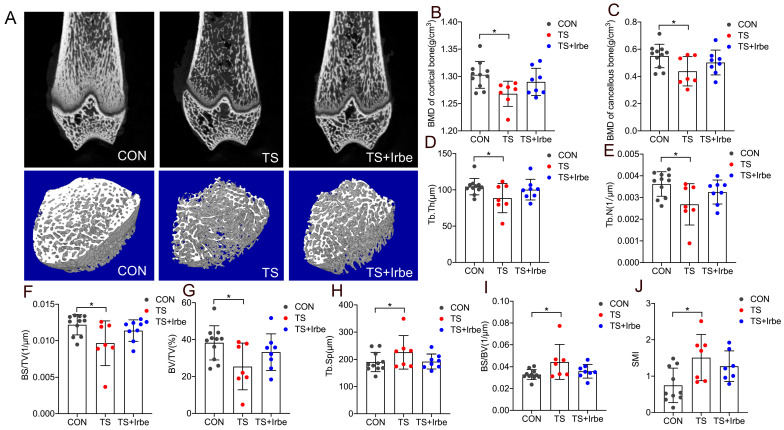
**Inhibition of AGEs partially improved bone microstructure after tail suspension.** (**A**) 2D reconstruction images of distal femur and 3D images of region of interest of trabecula. (**B**) BMD of cortical bone. (**C**) BMD of cancellous bone. (**D**) Tb.TI (**E**) Tb.N. (**F**) BS/TV. (**G**) BV/TV. (**H**) Tb.Sp. (**I**) BS/BV. (**J**) SMI. BMD: bone mineral density; Tb.Th: trabecular thickness; Tb.N: trabecular number; BS/TV: bone surface per tissue volume; BV/TV: bone volume per tissue volume; Tb.Sp: trabecular separation; BS/BV: bone surface per bone volume; SMI: structural model index. Values are all expressed as mean ± SD. * *p* < 0.05.

The bone microstructure parameters measured by Micro-CT are shown in Figure 3. The results showed that the BMD of cortical (Figure 3B) and cancellous bone (Figure 3C), the trabecular Tb.Th (Figure 3D), Tb.N (Figure 3E), BS/TV (Figure 3F), and BV/TV (Figure 3G) decreased significantly after tail suspension (*p* < 0.05), whereas a upward trend with the drug treatment in TS + Irbe group was observed; the trabecular Tb.Sp (Figure 3H), BS/BV (Figure 3I), and SMI (Figure 3J) increased significantly in the TS group compared to the CON group (*p* < 0.05), whereas a downward trend in TS + Irbe group compared to TS group was observed. These results suggested that bone microstructure of tail-suspended rats was partially improved with irbesartan treatment.

### 2.4. PEN Content Was Related to Bone Microstructure of Cancellous Bone

Further, we analyzed the correlation between bone microstructure parameters and the content of PEN at the corresponding location of micro-CT detection in the right distal femur. The results (Table 1) showed PEN of cancellous bone was negatively correlated with trabecular BMD (R = −0.485, *p* = 0.041), Tb.N (R = −0.519, *p* = 0.028), and BS/TV (R = −0.609, *p* = 0.009), and positively correlated with SMI (R = 0.505, *p* = 0.032), whereas it had negative trend of correlation with BV/TV (R = −0.482, *p* = 0.05) and had no correlation with Tb.Th, Tb.Sp, and BS/BV. However, PEN of cortical bone had no relationship with cortical BMD (R = −0.114, *p* = 0.6232).

### 2.5. Inhibition of AGEs Improved Bone Micromechanical Properties after Tail Suspension

**Figure 4:**
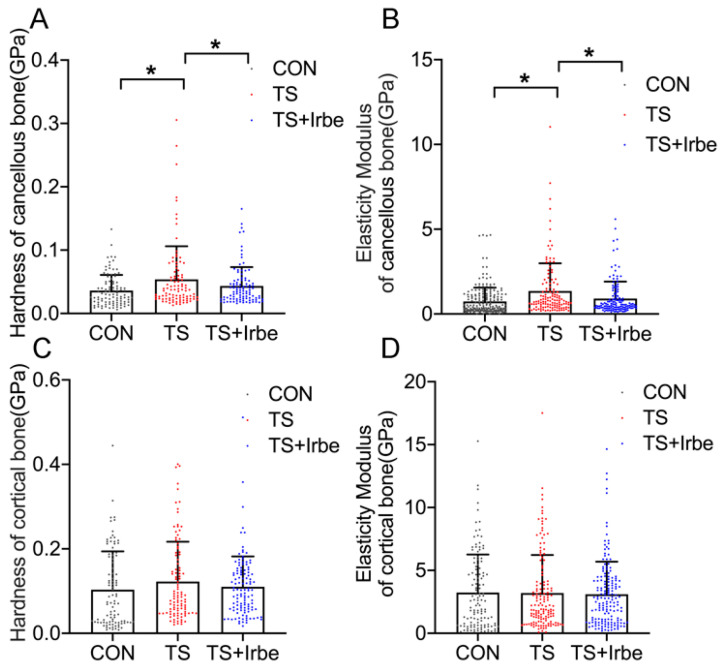
**Inhibition of AGEs improved bone micromechanical properties after tail suspension.** (**A**) Hardness of cancellous bone. (**B**) Elasticity modulus of cancellous bone. (**C**) Hardness of cortical bone. (**D**) Elasticity modulus of cortical bone. Values are all expressed as mean ± SD. * *p* < 0.05.

The bone micromechanical properties detected by nanoindentation are shown in Figure 4. The results showed that the hardness (Figure 4A) and elasticity modulus (Figure 4B) of the cancellous bone were obviously higher (*p* < 0.05), whereas both of them decreased significantly in TS + Irbe group compared to TS group (*p* < 0.05). However, the hardness (Figure 4C) and elasticity modulus (Figure 4D) of cortical bone showed no significant difference in TS and TS + Irbe groups compared to the CON group. The results suggested that bone micromechanical properties of tail-suspended rats were significantly improved with irbesartan treatment.

### 2.6. NE−xLR Was Related to Bone Micromechanical Properties in Cancellous Bone

Because bone micromechanical properties were proved to be directly influenced by the crosslinking structures of AGEs [39], in the present paper we investigated the relationship between non-enzymatic cross-links (NE−xLR) and bone micromechanical properties under tail suspension and irbesartan treatment via combining FTIR and nanoindentation at the same detection location. The results (Table 2) showed that NE−xLR in cancellous bone was positively correlated with hardness (R = 0.686, *p* < 0.0001) and elastic modulus (R = 0.547, *p* = 0.0031), whereas NE−xLR in cortical bone had no correlation with both hardness and elastic modulus.

### 2.7. Inhibition of AGEs Partially Improved Bone Metabolism after Tail Suspension

**Figure 5:**
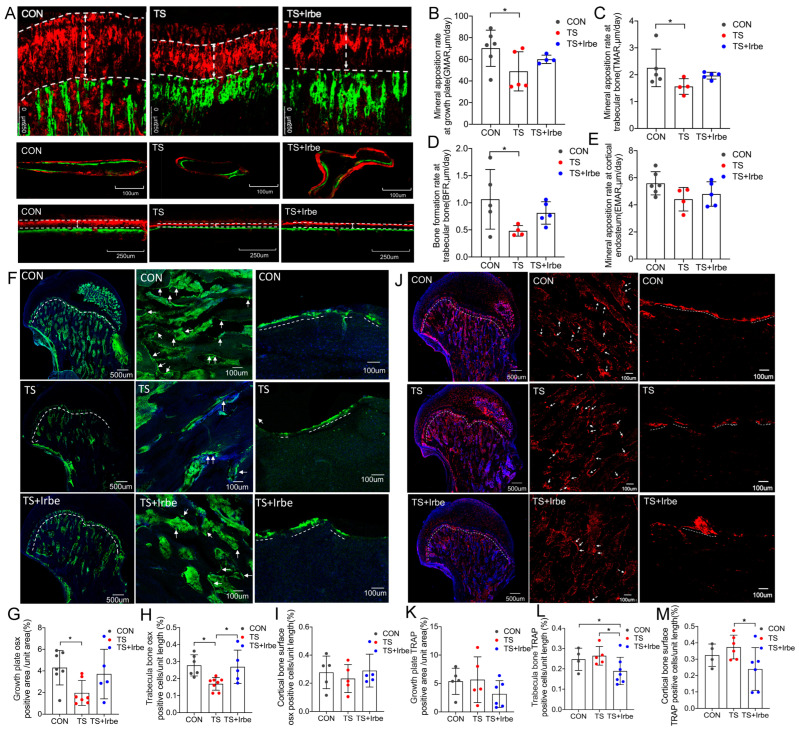
**Inhibition of AGEs partially improved bone metabolism after tail suspension.** (**A**) Representative images of calcein and xylenol orange double labeling at growth plate, trabecular bone, and cortical bone. Scale bar: 250 μm/100 μm. The green line was the calcein deposited in the newly formed bone, and the red line was the xylenol deposited in the newly formed bone. (**B**) Mineral apposition rate at growth plate (GMAR, μm/day). (**C**) Mineral apposition rate at trabecular bone (TMAR, μm/day). (**D**) Bone formation rate at trabecular bone (BFR, μm/I). (**E**) Mineral apposition rate at cortical endosteum (EMAR, μm/day). (**F**) Representative images of Osterix immunofluorescence staining in growth plate, trabecular bone, and cortical bone. White arrows and dashed lines indicate the location of positive cells (green), Nuclei was stained with DAPI (blue). Scale bar: 500 μm/100 μm. (**G**) Osterix positive area per unit area in growth plate (%). (**H**) Osterix (+) cells per unit length in trabecular bone (%). (**I**) Osterix (+) cells per unit length in cortical bone surface (%). (**J**) Representative images of TRAP immunofluorescence staining in growth plate, trabecular bone, and cortical bone. White arrows and dashed lines indicate the location of x cells (red), Nuclei was stained with DAPI (blue). Scale bar: 500 μm/100 μm. (**K**) TRAP positive area per unit area in growth plate (%). (**L**) TRAP (+) cells per unit length in trabecular bone (%). (**M**) TRAP (+) cells per unit length in cortical bone surface (%). Values are all expressed as mean ± SD. * *p* < 0.05.

Dynamic parameters of bone formation were assessed following in vivo calcein and xylenol orange staining. The results showed (Figure 5A–E) that mineral apposition rate (MAR) both at growth plate and trabecula bone decreased significantly in the TS group, as well as bone formation rate (BFR) at trabecular bone in the TS group compared to the CON group. All parameters showed an upward trend in the TS + Irbe group compared to the TS group.

The activities of osteoblastic and osteoclastic cells were detected by immunofluorescence staining with Osterix and TRAP. Osterix staining results showed that Osterix (+) cells in growth plate and trabecular bone (Figure 5G,H) decreased significantly (*p* < 0.05) in the TS group compared to the CON group, whereas in trabecular bone they increased significantly in the TS + Irbe group compared to the TS group. However, Osterix (+) cells in cortical bone had no significant difference in the TS and TS + Irbe group compared to the CON group (Figure 5I).

TRAP staining results showed (Figure 5J–M) that TRAP (+) cells in growth plate, trabecular bone, and cortical bone had no significant difference in the TS group compared to the CON group, whereas in trabecular bone and cortical bone they decreased significantly in the TS + Irbe group compared to the TS group (*p* < 0.05).

These results suggested that irbesartan increased osteoblastic cells activities but decreased osteoclastogenesis to partially improve bone metabolism in tail-suspended rats.

### 2.8. Fluorescent AGEs in Bone Matrix Was Related to Bone Metabolism Biomarkers

Furthermore, we investigated the relationship between fAGEs and Osterix/TRAP (+) cells under tail suspension and irbesartan treatment. The results (Table 3) showed that fAGEs in cancellous bone were negatively correlated with Osterix (+) cells (R = −0.661, *p* = 0.005), whereas no correlation with TRAP (+) cells was observed. Moreover, fAGEs in cortical bone were positively associated with TRAP (+) cells (R = 0.554, *p* = 0.026), whereas no correlation with Osterix (+) cells was observed.

## 3. Discussion

In this study, we mainly investigated the improvement effects of blocking AGEs formation on bone microstructure, bone micromechanical properties, and bone remodeling process of tail-suspended rats through the use of irbesartan, which is a kind of AGEs formation inhibitor. Our study showed that firstly, AGEs accumulated in the cancellous bone matrix and were involved in the deterioration of bone microstructure, bone micromechanical properties, and bone remodeling process under simulated microgravity, and secondly, that blocking AGEs formation by irbesartan can partially prevent the aforementioned disused bone loss.

First of all, we demonstrated the simulated microgravity effect had a negative impact on bone at multi-levels. The micro-CT results showed the notable deterioration of trabecular bone density and microstructure after tail suspension, as evidenced by decreased BMD, Tb. Th, Tb.N, BS/TV, and BV/TV, and increased BS/BV, Tb.Sp, and SMI (Figure 3C–G). Nanoindentation results revealed the hardness and elastic modulus of decalcified cancellous bone increased significantly after tail-suspension (Figure 4A,B), suggesting an abnormal increase in the stiffness and fragility of the organic phase (mainly collagen fiber) in the bone matrix. Moreover, the results of dynamic histomorphology revealed a reduction in dynamic bone formation, as indicated by lower values for MAR and BFR, in both the growth plate and trabecular bone following tail suspension (Figure 5A–E), which were consistent with the findings of Macias and Tian et al. [40,41]. As for the bone cells activities, Osterix as the transcription fact for osteoblasts differentiation in both growth plate and trabecular bone significantly increased (Figure 5F–I), whereas the number of TRAP (+) cells in growth plate and trabecular bone only showed a downward trend after tail suspension (Figure 5J–M), revealing the lower MAR and BFR in tail-suspended rats may be due to the inhibited differentiation of osteoblastic cells but unrelated to osteoclastic cells. Although inconsistent with the results of ground-based animal studies, our results were consistent with the findings in Sprague–Dawley rat flight studies [42,43,44]. Moreover, we found only the BMD decreased, whereas the mechanical properties, MAR/BFR, and TRAP (+)/Osterix(+) cells in cortical bone had no significant changes under simulated microgravity. This indicated that cancellous bone is more susceptible to be affected by simulated microgravity than cortical bone.

In order to investigate the essential factors of bone deterioration under simulated microgravity, we focused on the non-enzymatic glycation in the bone matrix and evaluated three kinds of matrix AGEs in bone from multi-points, including the overall content of PEN in bone, the 2D spatial distribution of crosslink AGEs (NE−xLR), and fluorescent AGEs (fAGEs). These global and microscopic measurements for AGEs not only provided detailed information on the changes in AGEs, but also help to further establish their connections with alterations of bone at different levels. The results found PEN (Figure 1A,B), NE−xLR (Figure 1C,D), and fAGEs (Figure 1E–G) all increased in cancellous bone but not in cortical bone after tail suspension, revealing the significant AGEs accumulation under simulated microgravity, which may be caused by the elevated serum glucose [8]. Meanwhile, the AGEs accumulation is more preferred to occur in cancellous bone first. This phenomenon is similar to bone deterioration under simulated microgravity, which occurred in cancellous bone prior to cortical bone. The reason may be that cancellous bone has a higher surface area per unit volume and a greater rate of metabolic activity compared to cortical bone, so the sugars in cancellous bone may be more easily accessible to matrix proteins and more likely to form AGEs [45,46].

Therefore, we then try to demonstrate that the elevated matrix AGEs played an essential role in the deterioration of bone under simulated microgravity. PEN was an accessible surrogate marker of AGEs in bone [38], and in this study, it was detected at the same anatomical locations with the bone microstructure parameters. Therefore, we analyzed the correlation between PEN and bone microstructure. The results found that accumulated PEN in the trabecular bone matrix is associated with less trabecular BMD, as well as sparser and more rod-like trabecular architecture, independently of cortical BMD (Table 1). These results suggested that the accumulated matrix AGEs can deteriorate the microstructure of the trabecular bone under simulated microgravity, which was similar to the findings in the study on diabetic osteoporosis [21].

Because the crosslinking AGEs formed on collagen can directly stiffen the collagen to reduce bone toughness [15], the elevated NE−xLR (the crosslinking structure AGEs) under simulated microgravity may be the reason for the alteration of bone mechanical properties. Thus, we analyzed the relationship between NE−xLR and the mechanical properties of the decalcified bone matrix via colocalizing the detection sites of FTIR and nanoindentation. According to the results, NE−xLR in cancellous bone but not in cortical bone was found to have a positive correlation with both hardness and elastic modulus (Table 2). Previously studies have not directly investigated AGEs effects on the stiffness of decalcified bone in vivo. It was only found that in vitro, the increased stiffness of decalcified human tibiae bone was related to the accumulation of bone matrix AGEs after incubating with ribose [47], and the nano-scale elastic modulus of cartilage matrix also increased significantly after L-threose treatment [48], suggesting in vitro glycation can stiffen the bone matrix to some extent. From our results, it was demonstrated that the accumulated in vivo matrix AGEs are the vital cause of the pathological stiffening of the cancellous bone matrix under simulated microgravity.

Furthermore, fAGEs can evaluate most kinds of AGEs in bone [28,49], and their microscopic distributions were observed in which Osterix/TRAP (+) cell activities were detected in our study. Hence, we analyzed the correlation between fAGEs and bone metabolism indicators. The results showed that fAGEs in cancellous bone were negatively related to Osterix (+) cells (Table 3), revealing matrix AGEs negatively impacted on the differentiation of osteoblasts under simulated microgravity. This is similar to our previous study, which found elevated matrix AGEs in vivo suppressed bone formation activities [50]. Besides, previous in vitro studies also reported AGEs can downregulate Osterix, osteocalcin (OCN), and collagen Ialpha1 (COL Iα1) expression to suppress osteoblast growth and differentiation [22,25,51]. Surprisingly, though both fAGEs and TRAP in cortical bone had no significant changes after tail suspension, the two showed a moderate correlation. Although previous studies were controversial about the role of AGEs in the modulation of osteoclast activity [40,41,42,43], our results were also not sufficient to determine the relationship between AGEs and osteoclast activity under simulated microgravity. This may be because of the sample size, and more samples may be needed in future.

Above all, we can infer that the accumulated matrix AGEs in cancellous bone indeed contributed to worsening cancellous bone quality and the bone formation process under simulated microgravity; therefore, we applied irbesartan to inhibit AGEs accumulations in tail-suspended rats. Our results showed AGEs accumulation in cancellous bone in tail-suspended rats was inhibited by irbesartan treatment (Figure 1A–G). Simultaneously, 8-OHdG levels, a kind of ROS marker, in the bone matrix of tail-suspended rats were suppressed after irbesartan treatment (Figure 2A,B) as well. We inferred the reduced AGEs accumulation under simulated microgravity may be because irbesartan can decrease the ROS level in cancellous bone to suppress hydroxyl radical formation, further suppressing the autoxidation of sugars to dicarbonyl precursors of AGEs in order to block AGEs formation.

Further, we found inhibition of AGEs by irbesartan treatment can improve bone quality under simulated microgravity. The BMD and microstructure of the bone were partially promoted (Figure 3A–J), and the hardness and elastic modulus of the cancellous bone matrix were reduced in tail-suspended rats (Figure 4A,B). Meanwhile, the number of Osterix (+) cells and MAR/BFR in cancellous bone in tail-suspended rats were promoted (Figure 5A–I). On one hand, these results indicated irbesartan can attenuate AGEs-induced damage in osteoblast differentiation at the cellular level to consequently affect dynamic bone formation. On the other hand, AGEs are directly formed on collagen and may disturb the normal post-translational modification process of collagen and the enzymatic cross-links formation, so that their accumulation may suppress mineral deposition [52]. Therefore, our study indicated the inhibition of AGEs formation may promote mineralization and bone formation through improving collagen structure at tissue level under simulated microgravity. However, more investigations are also needed for the direct effects of AGEs on mineralization in future.

Unexpectedly, although we did not find the alteration of osteoclastic cell activities under simulated microgravity, we found irbesartan can significantly inhibit fAGEs and osteoclastogenesis in both trabecular and cortical bone after tail-suspension (Figure 5J–M). Previous studies found angiotensin II (AngII) can promote the expression of Receptor Activator of Nuclear Factor-κ B Ligand (RANKL) and vascular endothelial growth factor (VEGF) in osteoblast to promote osteoclast formation, and ARBs can suppress AngII production to inhibit osteoclastogenesis [53,54]. Hence, suppression of irbesartan, as a kind of ARBs, on osteoclast activity may not be through blocking AGEs formation but may be via inhibiting the formation of AngII under simulated microgravity, which needs to be further studied in the future.

In summary, our study confirmed that AGEs preferred to accumulate in the cancellous bone matrix and can adversely affect bone tissue at multi-levels including impairing bone microstructure, bone micromechanical properties, and bone formation process under simulated microgravity. However, irbesartan can suppress AGEs formation in tail-suspended rats via reducing reactive oxygen species to reduce dicarbonyl compounds generation. The inhibition of AGEs then partially alleviated the deterioration of bone quality and bone remodeling process to prevent disused bone loss. The findings in this study may provide a new clue for exploring the mechanism of microgravity-induced osteoporosis and finding effective countermeasures.

## 4. Materials and Methods

### 4.1. Animal Care and Experimental Designs

Eight-week-old female Sprague–Dawley rats weighing 200–230 g were obtained from the Vital River Laboratory Animal Technology Co. (Beijing, China). The housing unit of the animal facility was maintained at 25 ± 2 °C with a reversed 12/12 h light–dark cycle. Each rat was fed separately in a tail box. The rats were given regular chow and water ad libitum. Twenty-four female Sprague–Dawley rats were randomly divided into three groups (n = 8, each group): the control group (CON), the tail suspension group (TS), and the tail suspension group treated with irbesartan (TS + Irbe). In TS and TS + Irbe groups, rats were elevated to produce a 30° head-down tilt for a duration of 21 days as mentioned in our work previously [55], which simulated the weightlessness of hindlimbs. In addition, rats in the TS + Irbe group were extra administered irbesartan at a concentration of 50 mg/kg/day. Calcein (5 mg/kg) and Xylenol orange (90 mg/kg) were administered 10 and 3 days prior to the end of the study to label actively forming bone surfaces.

**Figure 6:**
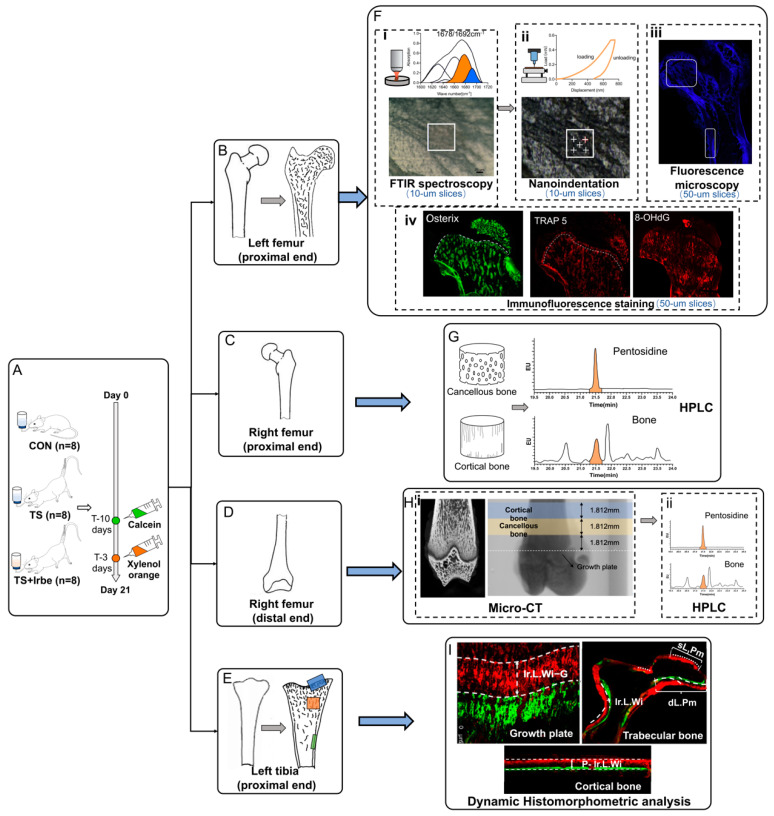
**Schema illustrating key aspects of the experimental procedure.** (**A**) Calcein (5 mg/kg) and Xylenol orange (90 mg/kg) were administered 10 and 3 days prior to the end of the study to label actively forming bone surfaces. After sacrifice, the proximal ends of left femur in rats were decalcified, embedded, and cut into frozen slices (**B**), and NE−xLR was analyzed by FTIR (**Fi**); bone micromechanical properties were determined by nanoindentation (**Fii**), the box indicates the detection locations of FTIR and nanoindentation (100 μm×100 μm); fluorescent AGEs were observed by autofluorescence microscopy (**Fiii**); the expression of Osterix, TRAP, and 8-OHdG were analyzed by immunofluorescence staining (**Fiv**). The proximal ends of right femur were divided into cancellous and cortical bone (**C**), and PEN content in bone matrices were determined by HPLC (**G**). The distal ends of right femur (**D**) were scanned and reconstructed to analyze bone microstructure by Micro-CT (**Hi**), and PEN content of corresponding sites of cancellous bone and cortical bone were also determined by HPLC (**Hii**). The proximal ends of left tibia were embedded and cut into slices for dynamic histomorphometric analysis (**E**), and the MAR/BFR at growth plate (blue diamond), trabeculae (orange diamond) and cortical endosteum (green diamond) were measured (**I**).

After that, the rats were sacrificed for subsequent detection. The unloaded hindlimbs of rats were our research objects. Firstly, the proximal femur has a femoral head allowing better separation of trabecular bone tissues, so that was chosen to detect AGEs content and bone indicators at the corresponding anatomical location. The left proximal femurs were used to successively detect the non-enzymatic cross-link (NE−xLR) by Fourier Transform infrared spectroscopy (FTIR), the fluorescent AGEs(fAGEs) by fluorescence microscopy, the bone micromechanical properties by nanoindentation, and the Osterix, TRAP and 8-OHdG expressions by immunofluorescent staining. The proximal ends of the right femurs were used to detect the pentosidine (PEN) content by High Performance Liquid Chromatography (HPLC). Then, the distal femur was found to have relatively rich trabeculae, so that its bone microstructure parameters had an overall representativeness. Thus, the distal femur was used to detect bone microstructure by micro-CT and the local PEN content at the same detected sites by HPLC. Finally, the left proximal tibiae were used to detect the mineral apposition rate (MAR)/bone formation rate (BFR) by dynamic histomorphometric analysis. Key experimental procedures are summarized in Figure 6, and the following are the details on the materials and methods used in this study.

### 4.2. Sample Preparation

The proximal end of the left femur was fixed in 4% paraformaldehyde for 48 h and decalcified in 0.5 M EDTA (pH 7.4) for 10d. Then, the decalcified bones were immersed in 20% sucrose and 2% polyvinylpyrrolidone (PVP) solution for 48 h to dehydrate. The tissues were finally embedded and cut into 10 μm thick or 50 μm thick longitudinal slices (coronal plane) by using a freezing microtome (Leica CM1950, Germany). The right femurs were stored at −80 °C.

The proximal end of the left tibia was placed in a gradient of ethanol (70%, 75%, 80%, 95%, and 100%) for dehydration and embedded in epoxy resin glue (A: B = 2:1) for 7 days, and then was cut into 50 μm thick longitudinal slices along a coronal plane by using a hard-tissue microtome (PRESI Mecatom T180, France).

### 4.3. Non-Enzymatic Cross-Links in Bone Matrix Determination by FTIR

FTIR imaging and spectra collection of 10 μm thick femoral slices was carried out using a Fourier transform infrared spectrometer equipped with a microscope (Shimadzu AIM-9000, Kyoto, Japan). Several locations (i.e., 7–8 locations) in cancellous and cortical bone were randomly selected for the test. For every location, spectra were acquired at a 100 μm×100 μm pixel size and in the reflective mode over a spectral range of 500 to 3500 cm^−1^ wave numbers at a resolution of 2 cm^−1^ with 16 scans per pixel. Then, the spectra were extracted by the AIM solution software (Version 1.1.0.0, Shimadzu, Japan,) and smoothed by IR solution software (Version 2.20, Shimadzu, Japan) with 10 smoothing points. Because the amide I band possesses structural information about the collagen matrix, it was targeted (1600–1710 cm^−1^) as this band [56] and fit with seven Gaussian components set at 1610, 1630, 1645, 1661, 1678, 1692, and 1702 cm^−1^ by Origin software (Version 2018, Origin Pro2018, Originlab) as previously reported [57]. Among them, the 1678 cm^−1^ absorption is associated with the β-turn conformation of collagen, which is a binding residue required for cross-links between two collagen molecules [58], and the 1692 cm^−1^ serve as a measure of collagen content [56]. Thus, the 1678 cm^−1^-to 1692 cm^−1^ ratio [57] was calculated to obtain non-enzymatic cross-links (NE−xLR) in the bone matrix (Figure 1(Fi)).

### 4.4. Bone Micromechanical Properties Determination by Nanoindentation

Following FTIR determination, five indentations were performed in the diaphragm at low/high NE−xLR locations relatively in cancellous and cortical bone in the same slices with FTIR, for one slice per rat. Two 2×2 matrices, respectively, for cancellous and cortical regions of each slice (a total of 384 indents among three groups) were randomly selected to perform nanoindentation using a nanoindentation tester (Nano Indenter G200, Agilent, Santa Clara, CA, USA) equipped with a Berkovich diamond tip. The tip was lowered with a loading rate of 10 nm/s to a maximum depth of 700 nm, held for 30 s, and subsequently unloaded. The resulting load-displacement curves were analyzed for hardness (Equation (1)) and elastic modulus (Equation (4)) using the Oliver–Pharr method [59].

(1) $$H=\frac{P_{\max}}{A}$$

(2) $$S=\frac{\mathrm{dP}}{\mathrm{dh}}$$

(3) $$E_{r}=\frac{\sqrt{\pi }S}{2\sqrt{A}}$$

(4) $$\frac{1}{E_{r}}=\frac{1-v^{2}}{E}+\frac{\left(1-v_{i}{}^{2}\right)}{E_{i}}$$

where $P_{\max}$ is the maximum load, A is the contact area, S is the slope of the 95–40% region of the unloading curve, $E_{r}$ is the equivalent elastic modulus, v is the Poisson’s ratio of bone (0.3) [60], $E_{i}$ and $v_{i}$ are the elastic modulus and Poisson’s ratio of the diamond indenter (1140 GPa and 0.07, respectively). (Figure 1(Fii)).

### 4.5. Fluorescent AGEs Observation by Autofluorescence Microscopy

The spontaneous fluorescence intensity of 50 μm thick left femoral slices was examined using a fluorescence microscope (Olympus DP80, Shinjuku City, Japan) with an excitation filter of 365 ± 5 nm and a barrier filter of 400 nm ± 35 nm as before [28]. The mean fluorescence intensity in cancellous and cortical regions of bone was, respectively, quantified using Image J software (Version 1.8.0, National Institute of Health, Bethesda, MA, USA) (Figure 1(Fiii)).

### 4.6. Bone Metabolism Biomarkers Analysis by Immunofluorescence Staining

Following the nano-indentation, immunofluorescence staining of 50 μm thick proximal femur slices was performed as previously described [61]. Briefly, after treatment with 0.3% Triton (9002-93-1, Solarbio, Beijing, China) for 1.5 h, slices were blocked with 4% Bovine serum albumin (BSA) at room temperature for 2 h and incubated overnight at 4 °C with primary antibodies: Osterix (ab209484, Abcam, Cambridge, UK, 1:400), TRAP (bs-16578R, Bioss, Beijing, China, 1:800), and 8-OHdG (sc-393871, Santa Cruz, CA, USA, 1:50). Then, secondary antibodies were incubated at room temperature for 2 h to visualize primary antibodies: Cy3-labeled Goat Anti-Rabbit IgG (A0516, Beyotime, Shangahi, China, 1:400) and Alexa Fluor 488-labeled Goat Anti-Rabbit IgG (A0423, Beyotime, China, 1:400). Finally, the slices were mounted with mounting media containing DAPI (36308ES20, Yeasen, Shangahi, China). A laser confocal microscope (CM1950, Leica, Wetzlar, Germany) was used for observation. Positive cells per unit area in the growth plate, and positive cells per unit length around trabeculae and cortical bone were calculated by Image J software (Figure 1(Fiv)).

### 4.7. Pentosidine (PEN) Content of Bone Determination by High Performance Liquid Chromatography (HPLC)

The pentosidine, a standard biomarker for AGEs in bone tissue [38], at different anatomic locations in the bone matrix was measured by HPLC. First, the proximal and distal ends of the femur were cut into pieces, and the bone trabecula tissue and cortical bone tissue from the right proximal femur and the micro-CT detected sites in the right distal femur were selected with the attached bone marrow carefully removed. Then, bone tissues were hydrolyzed in a sealed centrifugal tube, containing 5 mL of 6 M HCl, at 110 °C for 24 h. After that, 10 μL of the hydrolysate was extracted for hydroxyproline detection using Hydroxyproline Assay Kit (MAK008, sigma, St. Louis, MO, USA). The remaining part was concentrated by using a centrifugal concentrator (LaboGene ScanVac Maxi Vac, Allerød, Denmark), with 1500 r/min at 60 °C for 20 h, resuspended with 100 μL of distilled water.

The PEN content of these samples was quantified by a UHPLC system with a 2475 Multi λ fluorescence detector (ACQUITY Arc, Waters Corp, Milford, MA, USA), as previously described [62]. PEN was separated on Waters XSELECT HSS T3 column (4.6 × 100 mm, 2.5 μm) with a flow rate of 1 mL/min and a temperature of 40 °C, by using a gradient solution. Solvent A consisted 0.2% of trifluoroacetic acid (TFA) in acetonitrile, and solvent B consisted 0.2% of TFA in 18 Ω pure water. PEN was monitored for fluorescence at an emission of 378 nm and an excitation of 328 nm. The content of PEN was calculated based on a standard curve using standards (124505-89-7, Cayman, Ann Arbor, MI, USA) and normalized by the total amount of collagen (7.14 times hydroxyproline, pmol/mg) (Figure 1G).

### 4.8. Bone Microstructure Determination by Micro-CT Analysis

Micro-CT (Skyscan 1272, Belgium) was used to scan the distal end of the right femur. The 180 degrees of total rotation scanning was conducted, with increment angle of 0.6 degrees and scanning accuracy of 12 μm.

For analysis, a thickness of 1.812 mm was selected from both the cortical and trabecular regions of the distal femur, 1.812 mm distance away from the growth plate. Values for BMD were calculated, as well as microstructure parameters including trabecular thickness (Tb.Th, μm), trabecular number (Tb.N, 1/μm), percent bone volume (BV/TV, %), bone surface/tissue volume (BS/TV, 1/μm), trabecular separation (Tb.Sp, μm), specific surface (BS/BV, 1/μm), and Structure Model Index (SMI) (Figure 1(Hi)).

### 4.9. Dynamic Histomorphometric Analysis of Bone

The slices of proximal tibia were imaged using a laser confocal microscope (Leica CM1950, Germany). The mineral apposition rate at regions of growth plate, trabeculae, and cortical endosteum (GMAR, TMAR, EMAR, μm/d, Equations (5)–(7), as well as bone formation rate at trabecular bone (BFR, μm/day, Equation (9)) were measured and calculated by Image J.

(5) $$\mathrm{GMAR}=\frac{\mathrm{Ir}.L.{\mathrm{Wi}}_{-G}}{t}$$

(6) $$\mathrm{GMAR}=\frac{\mathrm{Ir}.L.{\mathrm{Wi}}_{-G}}{t}$$

(7) $$\mathrm{EMAR}=\frac{\mathrm{Ir}.L.{\mathrm{Wi}}_{-E}}{t}$$

(8) $$\mathrm{MS}/\mathrm{BS}=\frac{1/2\mathrm{sL}.\mathrm{Pm}+\mathrm{dL}.\mathrm{Pm}}{\mathrm{Tb}.\mathrm{Pm}}$$

(9) BFR=TMAR×MS/BS

where t is the time interval of the labeling periods (7 days), $\mathrm{Ir}.L.{\mathrm{Wi}}_{-G}$, $\mathrm{Ir}.L.{\mathrm{Wi}}_{-T}$, and $\mathrm{Ir}.L.{\mathrm{Wi}}_{-E}$ are the distances of double label at growth plate, trabeculae and cortical endosteum respectively, sL.Pm and dL.Pm are the single and double labeled perimeter at trabeculae, respectively, and Tb.Pm is the trabecular perimeter (Figure 1I).

### 4.10. Statistical Analysis

All data were analyzed using SPSS 19.0 software. The differences between groups were analyzed by a one-way ANOVA and followed by an LSD post hoc test. The correlation between NE−xLR and bone micromechanical properties, as well as that between fAGEs and bone metabolism markers were all analyzed by Pearson analysis. The data are presented as mean ± SD. The statistical significance was considered when *p* < 0.05.

## Figures and Tables

**Table 1:** Pearson correlation coefficient between PEN and bone microstructure parameters of cancellous bone (* *p* < 0.05).

	PEN of Cancellous Bone	
	R	*p*
BMD	−0.485	0.041 *
BV/TV	−0.482	0.050
Tb.Th	−0.367	0.148
Tb.N	−0.519	0.028 *
Tb.Sp	0.365	0.137
BS/BV	0.277	0.263
BS/TV	−0.609	0.009 *
SMI	0.505	0.032 *

**Table 2:** Pearson correlation coefficient between NE−xLR and bone micromechanical properties (* *p* < 0.05).

	NE−xLR in Cancellous Bone		NE−xLR in Cortical Bone	
	R	*p*	R	*p*
Hardness	0.686	<0.0001 *	0.135	0.548
Elastic modulus	0.547	0.0031 *	0.273	0.218

**Table 3:** Pearson correlation coefficient between fAGEs and bone metabolism (* *p* < 0.05).

	fAGEs in Cancellous Bone		fAGEs in Cortical Bone	
	R	*p*	R	*p*
Osterix (+) cells	−0.611	0.005 *	−0.187	0.5055
TRAP (+) cells	0.4414	0.0996	0.554	0.0260 *

## Data Availability

The data that support the findings of this study are available from the corresponding author upon reasonable request.
